# A Comparison between Chemical Synthesis Magnetite Nanoparticles and Biosynthesis Magnetite

**DOI:** 10.1155/2014/384984

**Published:** 2014-06-01

**Authors:** Seyed Abolghasem Kahani, Zahra Yagini

**Affiliations:** Department of Inorganic Chemistry, Faculty of Chemistry, University of Kashan, Kashan, Iran

## Abstract

The preparation of Fe_3_O_4_ from ferrous salt by air in alkaline aqueous solution at various temperatures was proposed. The synthetic magnetites have different particle size distributions. We studied the properties of the magnetite prepared by chemical methods compared with magnetotactic bacterial nanoparticles. The results show that crystallite size, morphology, and particle size distribution of chemically prepared magnetite at 293 K are similar to biosynthesis of magnetite. The new preparation of Fe_3_O_4_ helps to explain the mechanism of formation of magnetosomes in magnetotactic bacteria. The products are characterized by X-ray powder diffraction (XRD), infrared (IR) spectra, vibrating sample magnetometry (VSM), and scanning electron microscopy (SEM).

## 1. Introduction

In nature, the mineralized tissues, such as bone, teeth, diatoms, and shells, are produced by biological organisms. Biomineralization is the sophisticated process of production of these inorganic minerals by living organisms. The formation of magnetite (Fe_3_O_4_) particles by magnetotactic bacteria (MTB) is one example of a biomineralization process [1]. Magnetotactic bacteria are a diverse group of microorganisms with the ability to use geomagnetic fields in the direction sensing. The discovery of MTB stimulated interdisciplinary research interest among scientists, including microbiologists, physicists, geologists, chemists, and engineers. A characteristic of all MTB is the presence of magnetosomes in them. The magnetosome particles are used in the manufacture of magnetic targeting of pharmaceuticals, cell separation, and their application as contrast-enhancement agents in magnetic resonance imaging [2]. The magnetite particle distributions in the magnetosome typically are 35 to 120 nm. In general, the particle size distribution in the magnetosome is narrow, whereas a broader size range in some of the chemical synthesis grown crystals is common [3]. In MTB, the iron content per cell is in the range of 10^−13^ to 10^−14^ g and its estimates is 2% of the dry weight. In addition to Fe_3_O_4_, the cell contains ferrous ion, hydrous ferric oxide, and ferrihydrite. Moreover, bacterial magnetosome formation might serve as a model system for the biomineralization of magnetic minerals in other organisms, as similar crystals of ferromagnetic material, mainly magnetite, have been found in a wide range of higher organisms and even humans. Thus, an understanding of the structures and mechanisms involved in bacterial biomineralization of magnetosomes is of crucial interest [4]. There are several important questions about magnetite synthesis that need to be addressed. Magnetic biogenic minerals are produced by microbial activity in a wide range of subsurface environments. Magnetite is usually produced by both Fe(III) reducing and Fe(II) oxidizing bacteria, and understanding the formation of biogenic magnetite is particularly important [5]. In this paper, we propose a new chemical synthesis method for the preparation of magnetite from ferrous salt and using air as oxidizing agent in an alkaline aqueous solution at low temperature. Chemical methods for preparation of magnetite may be classified according to temperature. In the first dry process, the magnetite by reduction of hematite in the stream of H_2_ at high temperature (250–600°C) is produced [6]. In the second wet process, partial oxidation of Fe^II^ salt solution with KNO_3_ either takes place under an alkaline condition at low temperature (90°C) or is precipitated of a mixed Fe^II^/Fe^III^ solution with a mole ratio of 0.5. Recently, a new method in preparation of magnetite nanoparticle from iron oxyhydroxides (goethite, akaganeite, lepidocrocite, feroxyhyte, and ferrihydrite) and ferrous salt in aqueous solution was reported [7]. In the chemosynthesis of magnetite the amount of dissolved oxygen, pH, and temperature are important. Oxygen is an extremely reactive gas which vigorously oxidizes many elements at room temperature. Many inorganic compounds react directly with O_2_ under appropriate conditions [8]. Oxygen is an ideal oxidant in acidic or alkaline medium. It dissolves to the extent of 3.08 cm^3^ (gas at STP) in 100 cm^3^ H_2_O at 20°C and this drops to 2.08 cm^3^ at 50°C. Solubility of O_2_ in salt water is slightly less but is still sufficient for the vital support of marine and aquatic life. When iron corrodes in the presence of air, hydrogen is released, then water becomes alkaline, and ferrous ions are produced. In strongly aerated regions, the ferrous ions are oxidized to ferric ions and these ferric ions react with the hydroxyl to form a brown deposit of ferric hydroxide; in less aerated regions, the action of oxygen leads to the separation of magnetite. Whereas Fe(II) is more soluble than Fe(III) at neutral pH and it is easily taken up by bacteria. The new method of preparation of magnetite is an attempt to explain the formation of magnetosome.

## 2. Materials and Methods

### 2.1. Materials

All of the reagents were obtained from Merck Co. as analytical grade. All the reactions for the preparation of magnetite were carried out under the air blown atmosphere. The prepared magnetites were dried at room temperature in the form of powder. The samples (A1–A4) are characterized by X-ray powder diffraction (XRD), infrared spectra (IR), and vibrating sample magnetometry (VSM). XRD measurements were performed using a Philips X'pert pero MPD diffractometer with Cu K*α* radiation in the range 2*θ* from 10 to 80 at room temperature. IR was obtained as KBr pellet in the range from 4000 to 400 cm^−1^ using Shimadzu FTIR spectrometer. The vibrating sample magnetization (PAR-VSM155R) was used to evaluate the magnetic parameters.

### 2.2. Synthesis of Magnetite

The air was flushed through a 500 mL, two-necked, round-bottomed flask. The flask was charged with 5.96 g (0.03 mole) FeCl_2_·4H_2_O along with 150 mL deionized water and then 60 mL of 1 M NaOH was added while stirring vigorously. The reaction mixture is under the air blown for two hours at 273 K. During the reaction, the pH changed into the range of 8 to 9 and a black precipitate was formed. After precipitation is completed, the Fe_3_O_4_ particles were repeatedly washed with distilled water and then filtered and dried under vacuum at room temperature. Similarly, the reaction is carried out for preparation of magnetite samples (A2–A4) at temperatures 283, 293, and 303 K. The chemical reaction of Fe_3_O_4_ preparation can be described as follows:

(1) $$\begin{array}{c} 3\text{Fe}{\text{Cl}}_{2}+6\text{NaOH}⟶3\text{Fe}{\left(\text{OH}\right)}_{2}+6\text{NaCl}\\ 2\text{Fe}{\left(\text{OH}\right)}_{2}+0.5{\text{O}}_{2}⟶2\text{FeOOH}+{\text{H}}_{2}\text{O}\\ 2\text{FeOOH}+\text{Fe}{\left(\text{OH}\right)}_{2}⟶{\text{Fe}}_{3}{\text{O}}_{4}+2{\text{H}}_{2}\text{O} \end{array}$$

The yield, crystalline size, and maximum size distribution of samples (A1 to A4) are fully illustrated in Table 1.

## 3. Results and Discussion

**Figure 1:**
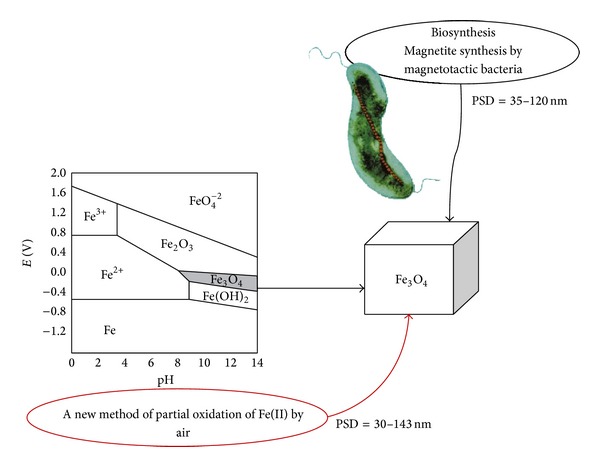
A schematic presentation pathway of chemical preparation of magnetite and biosynthesis of magnetite and their particles size distributions.

The mechanisms of magnetite formation by bacteria are still under investigation and have been studied most intensively. The process by which the magnetosomes are made and organized is not completely known [9]. The comparison of the new method in preparation of magnetite with magnetite formation in magnetotactic bacteria is important. It shows how the magnetosomes are formed in bacterial biomineralization. The reaction pathways of magnetosomes formation and new chemosynthesis of magnetite nanoparticle preparation are shown in Figure 1. The crystalline size, morphology, and particle size distributions of synthetic magnetites compared to the magnetite isolated from magnetotactic bacteria are different. The magnetosome particle sizes typically are from 35 to 120 nm. In general, the particle size distribution of magnetite in magnetosome is narrow, whereas a broader size range distribution is common in some chemosynthesis of magnetite methods [10, 11]. In new method, the particle sizes of chemical magnetites are in the range of that produced by magnetotactic bacteria. In sample A3, the crystal size distribution, morphology, and magnetic properties are also similar to the biomagnetite. In the preparation of magnetite, these properties are available when the reaction conditions are controlled. The potential-pH diagram is useful in the study of reactions in an aqueous solution. Pourbaix diagram has significant role in magnetite formation. Magnetite is produced in a slightly basic and reducing suboxic environment. The stability field of magnetite under normal conditions is relatively narrow because the magnetite exposed to the open air is prone to oxidation [12]. When the reaction conditions are controlled by using the Pourbaix diagram, a similarity between the new magnetite chemosynthesis formation and biosynthesis is available. According to the observation, it is proposed that the magnetite is produced by both Fe(III) reducing and also Fe(II) oxidizing process. The formation of this nanoscale mineral through the bioreduction of Fe(III) coupled to organic matter oxidation is well established and described in detail. Recent research suggests that magnetite can be formed via biological oxidation of Fe(II). The iron used in the formation of magnetosome comes from the Fe^+2^ or Fe^+3^ in the environment, but Fe^+2^, which is highly soluble and therefore easily uptaken, is preferred in the environment. The thermodynamic calculation showed that the magnetite synthesis from Fe(II) by molecular oxygen in aqueous solution is energetically favorable. MTB is aerotactic and tend to find the maintaining position at a preferred concentration of oxygen [13]. Subsequent adjustment of the redox potential and pH causes nucleation and growth of magnetite crystals. Thus, by means of them, it is possible to predict, on a thermodynamic basis, for a given Fe(II), the equilibrium states of all the possible reactions between ions and its solid and gaseous compounds in the presence of water. The mechanism of an aerobic Fe(II) oxidation is poorly understood, with important unanswered questions including the cellular location of Fe(II) oxidation. Magnetotactic bacteria can be regarded as a model for studying the structural, chemical, and magnetic properties of arrangements of ferrimagnetic iron oxide nanocrystals. It is evident that the reaction conditions are affected by the size, shape, crystal structure, crystallographic orientation, and crystal size distribution in new chemosynthetic magnetite. Therefore, it is necessary to optimize the amount of dissolved oxygen, pH, and temperature similar to the ecological living of magnetotactic bacteria. We present recent results obtained using X-ray powder diffraction (XRD), IR spectra, scanning electron microscopy (SEM), and vibrating sample magnetometry (VSM).

### 3.1. Analysis of Crystalline Phase

**Figure 2:**
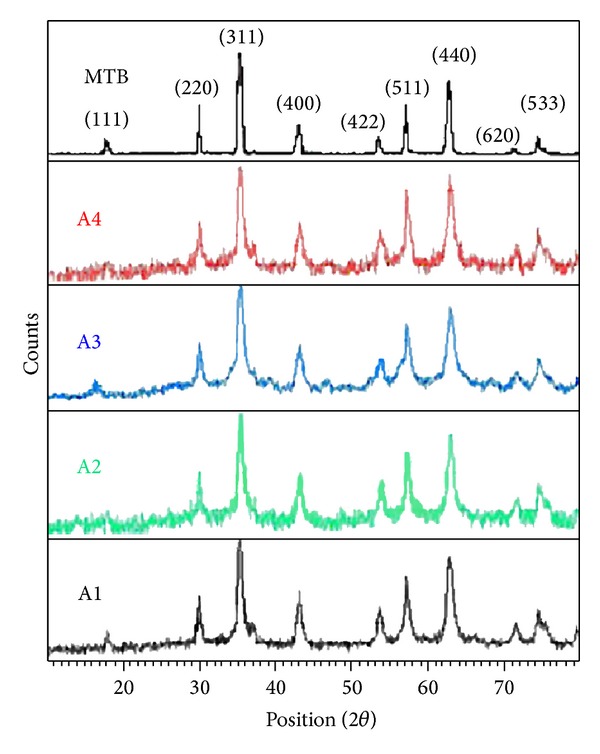
X-ray powder diffraction pattern of magnetite samples (A1 to A4) prepared at different temperatures compared to XRD pattern of magnetite in MTB was reported [9].

X-ray diffraction is a significant tool for the investigation of the fine structure of matter. Crystalline phase identification of synthetic magnetite was carried out by X-ray diffraction (XRD). As shown in Figure 2, the XRD pattern of synthetic magnetic nanocrystal and magnetosome matched well with the standard Fe_3_O_4_ reflections. Thus, we can confirm that both magnetic nanocrystal within the magnetosome and synthetic magnetic nanocrystal consisted of magnetites. The XRD pattern for all samples, which were prepared at 273 K (A1), 283 K (A2), 293 K (A3), and 303 K (A4), respectively, indicates that Fe_3_O_4_ is the dominant phase. Increasing the preparation temperature results in a remarkable broadening of the peaks that is due to decrease of crystalline size effect. The crystalline size could be estimated from the full width at half maximum (FWHM) of the diffraction peaks. The mean crystallite size was calculated from XRD line broadening using the Scherrer relationship [14]. The mean diameters of Fe_3_O_4_ crystallite size are 35.14, 23.23, 17.57, and 16.51 nm, respectively, for A1, A2, A3, and A4. Both synthetic magnetite and magnetosome had perfect crystallinity for their narrow FWHM. Thus, we can confirm that both magnetic nanocrystal within the magnetosome and synthetic magnetic nanocrystal consisted of magnetites. The comparison of crystalline size and maximum particle distribution of magnetite in synthetic samples with MTB was shown in Table 1. Crystallite size is a measure of the size of a coherently diffracting domain. Due to the presence of polycrystalline aggregates, crystallite size is not generally the same as the particle size. Crystallite size analysis on a sample containing extended defects can be used to estimate the ordered domain size (the size of the region between defects) in the same manner that XRD is used to determine crystallite size. However, the technique for measuring the particle size rather than the crystallite size is scanning electron microscopy (SEM). Both the synthetic magnetite and magnetosome had perfect crystallinity for their narrow FWHM. The magnetosome XRD patterns were all similar to those of synthetic magnetite in all diffraction directions. In general, the crystal size distribution in magnetosome is narrow, whereas a broader size range is common for chemical synthesis of magnetite. Here, the FWHM of magnetosome XRD patterns was similar to that of synthetic magnetite in all diffraction directions. Thus, it can be drawn that magnetosome synthesized by biomineralization had higher crystallinity similar to synthetic magnetite.

### 3.2. Infrared Spectroscopy

**Figure 3:**
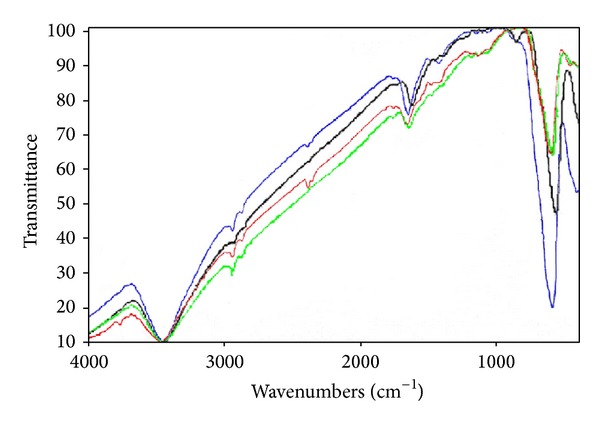
Infrared spectra of magnetite nanoparticles prepared at different temperatures: (blue A1) 273 K; (red A2) 283 K; (green A3) 293 K; and (black A4) 303 K.

The most widely applied technique used to identify and characterize iron oxide is infrared. For iron oxide, IR spectroscopy is useful as a means of identification. This technique also provides information about crystal morphology and hence the nature of surface hydroxyl groups and adsorbed water. Central to most of these studies is the use of vibration spectroscopy to determine the phonon density of states. There have been numerous studies of the vibration spectra of magnetite, with the majority employing infrared absorption and Raman scattering to assign the four T_1u_ infrared-active phonons and the A_1g_, E_g_, and 3T_2g_ Raman-active modes, respectively. The two most intense T_1u_ modes infrared-active of various magnetite samples were observed as two broad bands at 586 and 440 cm^−1^, 586 and 446 cm^−1^, 571 and 440 cm^−1^, and 578 and 440 cm^−1^, respectively, for A1, A2, A3, and A4 (Figure 3). The presence of magnetite nanoparticles is characterized by two strong absorption bands (T_1u_ modes) which are similar to the relevant Fe–O bond of bulk magnetite.

### 3.3. Magnetic Properties of Synthetic Magnetite

**Figure 4:**
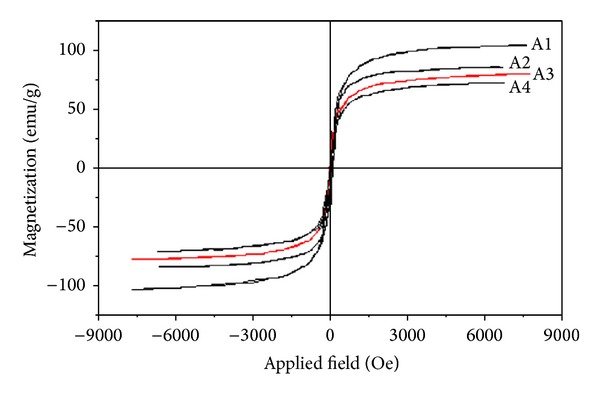
Magnetization versus applied field for magnetite nanoparticles prepared at different temperatures: (A1) 273 K; (A2) 283 K; A3) 293 K; and (A4) 303 K.

The magnetic properties of magnetite crystals, such as saturation magnetization, magnetic remanence, and coercive force, are largely controlled by domain state, which in turn depends upon grain size. The magnetization (*M*) versus field (*H*) curve at 300 K for samples of synthetic magnetite is shown in Figure 4. It was observed that synthetic magnetite (A3) had a magnetization of 78.2 emu/g, remanence of 12.9 emu/g, and coercivity of 98 Oe and shows a ferromagnetic behavior. In ultrafine magnetically ordered magnetite particles, the transition from superparamagnetic to ferromagnetic behavior occurs at a critical size (Dp of 25 nm) [15]. Thus, synthetic magnetites are ferromagnetic because their mean particle size is bigger than the critical size. The magnetosomes which were reported in literature also exhibited ferromagnetic behavior with a saturation magnetization of 60 emu/g, remanence of 10 emu/g, and coercivity of 75 Oe (Table 2). The magnetosomes are ferromagnetic because their mean size (45 nm) is larger than the critical size. The saturation magnetization of the synthetic magnetite (A3) is 78.2 emu/g, that is, larger than that of magnetosomes, 60 emu/g. However, the saturation magnetization value for magnetosome is significantly lower than synthetic magnetite, that is, due to the presence of lipid membrane. There is a nonmagnetic lipid membrane of 3–5 nm thickness in the outer of magnetosomes [16]. The results showed that both magnetosomes and synthetic magnetite (A3) have a similar magnetic response due to their perfect crystallinity.

### 3.4. Analysis of Particle Morphology and Particle Size Distributions

**Figure 5:**
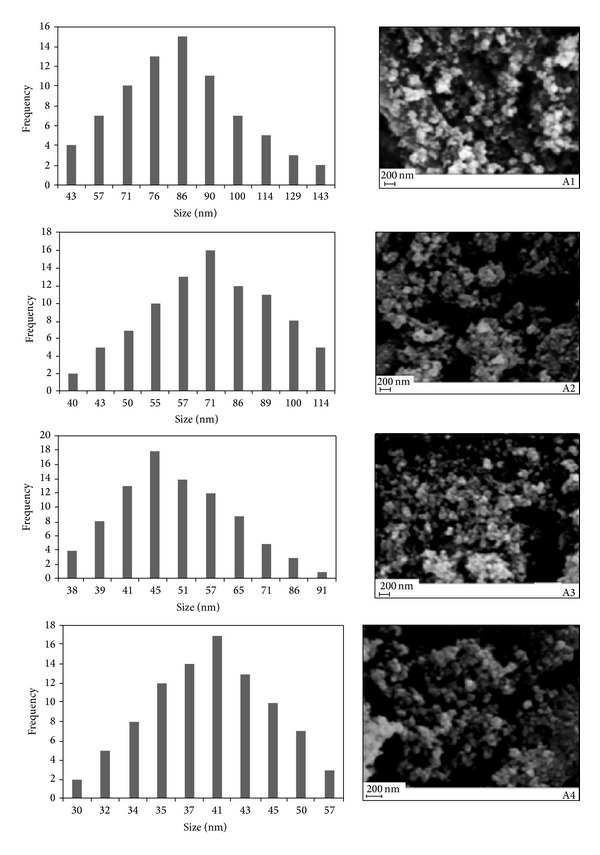
Scanning electron microscopic photographs of Fe_3_O_4_ nanoparticles prepared at different temperatures: (A1) 273 K; (A2) 283 K; A3) 293 K; and (A4) 303 K, and their particle size distributions.

The techniques for measuring the particle size and morphology of particles are transmission electron microscopy (TEM) and scanning electron microscopy (SEM). A series of studies on the magnetosomes are reported in the literature; the results show that the magnetite crystals have a narrow grain size distribution and also chemical purity. In general, the particle size distribution is narrow in biomagnetite and it varies from 35 to 120 nm, while its maximum frequencies are between 40–55 nm in the magnetosomes [17]. Statistical analysis of bacterial magnetite crystals from magnetosomes shows narrow asymmetric size distributions. The statistical analysis reflects the control of the bacteria, which limits the growth of the magnetite crystals to specific sizes and morphologies, as marked by the asymmetry of the size and shape distributions. Statistical analysis of sizes and shapes might provide robust criteria for distinguishing between biogenic and nonbiogenic magnetic crystals. The reasons of the synthetic magnetite could be distinguished from bacterial magnetite, despite the fact that the former was prepared by rapid precipitation from an aqueous solution, a process known to produce small crystals of homogeneous size distributions. In this paper, a new method in preparation of magnetite from Fe(II) and molecular oxygen in aqueous solution was proposed that the particle size of Fe_3_O_4_ is in the range of that produced by magnetotactic bacteria. The most similarity in crystal size distribution, morphology, and magnetic properties relative to the biomagnetite was observed in the sample A3. Here, the morphologies of synthetic magnetite were observed using a SEM. The particles (77, 89, 87, and 91) of synthetic magnetites were measured and plotted their frequencies as a function of particle size in samples (A1 to A4), respectively, as shown in Figure 5. We estimated the standard deviation according to the very large set of data equation. Here, the sum of squares of the individual deviations from the mean is divided by the total number of measurements in the set. Extraction of the square root of this quotient gives (*σ*_A1_ = 21.05, *σ*_A2_ = 21.13, *σ*_A3_ = 12.32, and *σ*_A4_ = 5.91), respectively. The results of the calculations show a normal distribution for A3 and A4. The particle size distribution of the synthetic magnetites (A3) varied from 38 to 91 nm, while its maximum frequencies are between 40–57 nm. Here, the particle size of magnetites is controlled by the concurrent oxidation of Fe(II) ion with molecular oxygen via the precipitation of that in the aqueous solution. From a thermodynamic point of view, in the presence of a neutral and alkaline pH and a low redox potential, the magnetite is favored when compared to those of other iron oxides.

## 4. Conclusion

Magnetites in nanoscale were synthesized by new chemical method. Particle size can be tuned by modifying reaction conditions such as dissolved oxygen, pH, and temperature. When the reaction temperature is increasing, it causes a decrease of particle size and monodisperse nanoparticles are obtained. In this present work, the amount of reactants, pH, and temperature are optimized similar to ecological living of magnetotactic bacteria. In chemical preparation of magnetite, we studied the effects of reaction conditions on the particle size and size distribution. The results show that the reaction temperature has a significant effect on them. The particle size and magnetic properties were also discussed through results obtained by using X-ray powder diffraction (XRD), IR spectra, scanning electron microscopy (SEM), and vibrating sample magnetometry (VSM).

## Figures and Tables

**Table 1:** The yield of magnetites prepared at different temperatures and their crystallite size and particle size distribution.

Sample	*T* (K)	FWHM (311)	Crystallite (nm) size (XRD)	Particle size distribution (SEM)	Yield (%)
A1	273	0.2362	35.1	43–143	86
A2	283	0.3542	23.2	40–114	95
A3	293	0.4723	17.6	38–91	97
A4	303	0.5314	16.5	30–57	93
MTB	—	0.471	17.6	35–120	—

**Table 2:** Magnetic properties of magnetite samples (A1 to A4) prepared at different temperatures and their comparison with magnetite in (MTB) bacteria.

Sample	*T* (K)	*M*_*s*_ (emu/g)	*B*_*r*_ (emu/g)	*H*_*c*_ (Oe)
A1	273	103.2	17.0	86.0
A2	283	85.0	22.7	103.0
A3	293	78.2	12.9	98.0
A4	303	71.5	12.6	77.5
MTB	—	60.0	10.0	75.0
